# Progress in Psychiatry

**Published:** 1903-05-16

**Authors:** 


### PROGRESS IN PSYCHIATRY.

(*Concluded from page* 101.)

**Alcoholism and Insanity.**—Alcoholism in the progenitors is a fruitful cause of insanity, idiocy, mental defect and nervous disease in the offspring. Alcoholism itself is of course frequently associated with mental disease in the family histories of insane persons, but for the purposes of inquiry into the special action of alcohol Dr. Wiglesworth[10] found on analysing 3,445 cases of insanity that a definite history of alcoholic excess unassociated with insanity, in one or both parents, was traceable in 578 instances. Separating the sexes it was found that 327 of these cases were males and 251 were females. "Doubtless some few of these cases of alcoholic excess may have been," adds Dr. Wiglesworth, "veritable examples of dipsomania . . . a neurosis allied to insanity, but as most of such cases usually show definite mental disorders at some period or other of their course, the majority of them will have been included in the tables of hereditary insanity referred to already in THE HOSPITAL, February 14th and 28th, 1903 (Progress in Psychiatry). These figures probably understated the actual frequency of alcoholic intemperance in the ancestry of the insane, for the friends of patients conceal or deny the presence of intemperance in the parentage, or otherwise deny that 'excess' in drink exists and admit that the limits of moderation have not been exceeded. The above figures therefore apply only to cases of gross and palpable alcoholic excess. In regard to the mode in which alcohol acts, we are concerned," adds Dr. Wiglesworth, "with a direct poisoning of the germ-plasma itself by means of the alcohol circulating in the blood, and consequent direct injury to the cells of which this structure is composed, which by reason of this injury are prevented from developing into a stable organism." Very dilute solutions of alcohol have been shown by Ridge[11] and others to be inimical to protoplasmic growth whether vegetable or animal, and when cells are in process of development they are more susceptible to morbific agents than when fully formed. A diffusible fluid like alcohol, acting thus on the sperm-cell, the ovum, or on the growing fœtus, might be expected to affect most the cells or organic elements which constitute the nervous system. A morbid character may thus become stamped upon either one of the germ or sperm-cells before the union of these two elements, and if the mischief is not counteracted by a healthy condition of the other of these two, the result will be that the development of the fœtus will be marked by lesions or defects from which there is no escape. If the alcoholic poisoning has reached a certain degree of intensity, idiocy or imbecility may be expected to result, "whilst if of less degree the injury may manifest itself in the various forms of adolescent or other insanity when adult life is developing or has been attained to."[12] If both parents are alcoholic, whatever injury may have been done to the germ-cell (spermatozoon) and ovum at impregnation will be added to and reinforced by the alcoholic poisoning of the nervous centres of the growing embryo during the whole or part of the period of intra-uterine life. An inquiry on a large scale into the subject of heredity and alcoholism was carried out recently in America by Dr. T. Crothers of Hartford, Connecticut, and a committee of physicians appointed along with him in 1888, of which he was chairman. The collection of material (clinical histories and cases) went on for 13 years, and the account of it, which was published by Crothers[13] recently, was based on fairly accurate histories of 1,744 cases of inebriety, the majority of which (about 1,300) had come under his personal care and observation. "The inquiry has not been confined to heredity alone, but has included every condition and circumstance which could have an ætiological bearing in the development of inebriety." The chief conclusion reached was that the injury produced by alcohol on the cells and nervous tissues was transmitted to the next generation with positive certainty in some form or other, *e.g.* as a neurosis, a cerebral (mental) defect, as a drink-craving, etc. Of 1,744 inebriates it was found that 1,080 had a distinct history of heredity—*i.e.* parental intemperance—in 390 cases the inebriety was traced to bodily injuries, diseases, or shocks for which alcohol was taken (as a remedy or otherwise), in 180 cases the inebriety was the sequel to starvation and poisoning, and in 85 cases it arose from ignorance, evil surrounding, and bad example. In only nine cases were the causes so obscure or complex that no classification could be made. In most of the 1,080 cases of inebriety with an heredity of the kind alluded to above it appeared that the patients showed a special predisposition to find relief in spirits, or a mental diathesis (instability) with loss of self-control, and often a species of psychical pain and unrest which found the greatest relief from the use of spirits. Four hundred and thirty of these patients showed a direct heredity (drink-craving in the parents), 224 showed an indirect or atavic heredity of the same kind, while 290 showed a psychopathic heredity in the parentage. While the family records showed some members with ability, and even brilliance, in theology, politics, music, law, etc., others were of criminal and vagrant tendencies or mentally defective and tending to lapse into the low strata of life. Forty-nine cases were grouped under the heading of "epileptoid" types of heredity in patients whose symptoms of alcoholism were of sudden and stormy development, not strictly or regularly periodic, "developing out of the clear sky in an impetuous storm and then disappearing and leaving little trace behind." The ancestors of this class of patients, says Dr. Crothers,[14] were "always noted by their sudden explosive nerve energy . . . persons whose whole conduct through life was an alternation of wild extremes . . . continually changing in politics, in religion, in business, and society . . . always leading forlorn hopes and always defeated, physically suffering from disease and making remarkable recoveries—at one time using spirits, then changing to other remedies, always under an obsession." Women who were the offspring of this ancestry were found to be neurotic subjects, addicted to drugs and to Christian science, faith curers and invalids of all grades and descriptions, often secret drinkers afflicted with a moral paralysis. Finally various groups were met with in which fatalistic tendencies to drinking (commencing at the same age at which their parents did) were observed, or which manifested precocious sexual instincts which would be gratified by any means whatever, and in a few cases there were observed abnormal cravings for food (gluttony), as well as for drink which never seemed to be satisfied. Finally, the morphia habit was observed in a good many instances.

**Mental Disorders simulating Neurasthenia.**—Professor E. Kraepelin, of Heidelberg,[15] states that owing to the current lack of precision in the use of medico psychological language the term neurasthenia has been frequently applied to the most varied forms of nervous disorders, and has often been misused as a designation for the initial stages of serious mental disorders before the precise nature of the latter could be apprehended. The differential diagnosis of neurasthenia from mental and cerebral diseases which may simulate it is therefore important. This applies especially to the early stages of general paralysis of the insane. The characteristic paretic speech (tremulous, embarrassed, and with slurring difficult sounds) and the oculo-papillary symptoms will, if present, serve to indicate general paralysis. The first indication of general paralysis may, however, be a maniacal outbreak which will serve to distinguish it positively from neurasthenia when diagnosis has been difficult. Some forms of dementia-praecox or mental enfeeblement supervening in adolescence are also liable to be confounded with neurasthenia, and this is especially so with the hebephrenic form of dementia præcox. The presence of masturbation, with its debilitating effects upon the central nervous system, may make the resemblance to neurasthenia more marked in the early stages of the psychoses of adolescence. "It is important to discriminate between neurasthenia and these psychoses of adolescence because useless and expensive efforts to obtain a cure, *e.g.*, by hydrotherapy, electricity, and hypnosis, may be avoided if it were known that the disease was dementia præcox and not neurasthenia, as only the latter could react favourably to such therapeutic measures. The development of such symptoms as stupidity and mental automatism, muscular hypertonicity and catalepsy, mannerisms, stereotyped attitudes and gestures, and negativism or resistance to every movement required —any one or two of these signs should lead us to suspect the presence of dementia præcox. Finally, there is a group of mental affections, such as maniacal-depressive insanity and circular insanity, which in the prodromal stages exhibit the appearance of neurasthenia. On inquiring into the psychical condition of these patients, however, there will be detected various symptomatic elements which, though present in neurasthenia, are found to be developed to a more intense degree in these mental cases, viz., great prostration with incapacity for work, a gloomy and clouded disposition, hypochondriacal apprehensions and fears, headache and cephalic paraesthesiæ, disagreeable sensations of various sorts about the body, feelings of weight in the limbs and joints, loss of sleep, and a continuous feeling of exhaustion. In cases of this class amelioration of symptoms occurs sooner or later, since the psychical condition fluctuates and does not present the steady persistence of the fatigue symptoms of neurasthenia. Neurasthenia is, as Möbius[16] puts it, a chronic idiopathic exhaustion of the nervous system, usually persistent throughout life. Neurasthenic patients get spurred on by friends and relatives to put forth efforts to "overcome the weakness," with the result that the reaction, in the form of fatigue, becomes more and more marked after such efforts. Many a neurastheniac is driven to the use of stimulants, such as alcohol or morphia. The latter drug is especially resorted to since its consumption is attended with feelings of well-being and heightened capacity for work lasting a few hours. The neurasthenic condition is associated with a deficiency of bodily metabolism, marked by an imperfect excretion of toxic waste-products and a defective capacity for recuperation of the nerve-cells even during rest and sleep. This condition presents points of analogy with myxœdema. Another group of cases of neurasthenia may be caused by sudden psychical disturbance produced by physical shock and traumatism. A sub-class of this group is composed of neurasthenias resulting from sudden fear, not necessarily accompanied by physical shock, and some of these are of grave prognosis. A large and important group of neurasthenias is the congenital form, and allied to this is a hypocondriacal variety, with predominant symptoms of anxiety, doubt, and scruple. These symptoms are closely related to the so-called "phobias" of mental disorder, such as agoraphobia, claustrophobia, and pathophobia. From time to time these symptoms of anxiety, doubt, and scruple acquire great intensity and invade the psychical sphere, giving rise to considerable mental perturbation, emotional disturbance, and even to delusions and impulsive insane acts. Kraepelin thinks that the term neurasthenia should be limited, so as to exclude psychopathic types which are marked by hypochondriacal symptoms, and by "phobias" generally, and believes that in this way a more discriminating diagnosis and a more individualistic treatment will be possible.

[10] Jour. of Ment. Science, Oct., 1902 (Presidential Address at the Medico-Psychological Association, by Dr. Wiglesworth. [11] Alcohol and Public Health. [12] Jour. of Ment. Science, Oct., 1902. [13] Quart. Jour. of Inebriety, Jan., 1901. [14] *Loc. cit.* [15] Münchener Medicinische Wochenschrift. No. 40, 1902. [16] Studien über-Neurasthenie, 1900.

